# Metabolic Changes of Cholangiocarcinoma Cells in Response to Coniferyl Alcohol Treatment

**DOI:** 10.3390/biom11030476

**Published:** 2021-03-22

**Authors:** Bundit Promraksa, Praewpan Katrun, Jutarop Phetcharaburanin, Yingpinyapat Kittirat, Nisana Namwat, Anchalee Techasen, Jia V. Li, Watcharin Loilome

**Affiliations:** 1 Department of Biochemistry, Faculty of Medicine, Khon Kaen University, Khon Kaen 40002, Thailand; bundit.p@kkumail.com (B.P.); jutarop@kku.ac.th (J.P.); yingpinn@gmail.com (Y.K.); nisana@kku.ac.th (N.N.); 2 Cholangiocarcinoma Research Institute, Faculty of Medicine, Khon Kaen University, Khon Kaen 40002, Thailand; anchte@kku.ac.th; 3 Center of Excellence for Innovation in Chemistry, Faculty of Science, Khon Kaen University, Khon Kaen 40002, Thailand; praewka@kku.ac.th; 4 Department of Chemistry, Khon Kaen University, Khon Kaen 40002, Thailand; 5 International Phenome Laboratory, Northeastern Science Park, Khon Kaen University, Khon Kaen 40002, Thailand; 6 Faculty of Associated Medical Science, Khon Kaen University, Khon Kaen 40002, Thailand; 7 Department of Metabolism, Digestion and Reproduction, Faculty of Medicine, Imperial College London, London SW7 2AZ, UK; jia.li@imperial.ac.uk

**Keywords:** coniferyl alcohol, metabonomics, cholangiocarcinoma

## Abstract

Cholangiocarcinoma (CCA) is a major cause of mortality in Northeast Thailand with about 14,000 deaths each year. There is an urgent necessity for novel drug discovery to increase effective treatment possibilities. A recent study reported that lignin derived from *Scoparia dulcis* can cause CCA cell inhibition. However, there is no evidence on the inhibitory effect of coniferyl alcohol (CA), which is recognized as a major monolignol-monomer forming a very complex structure of lignin. Therefore, we aimed to investigate the effect of CA on CCA cell apoptosis. We demonstrated that a half-inhibitory concentration of CA on KKU-100 cells at 48 h and 72 h was 361.87 ± 30.58 and 268.27 ± 18.61 μg/mL, respectively, and on KKU-213 cells 184.37 ± 11.15 and 151.03 ± 24.99 μg/mL, respectively. Furthermore, CA induced CCA cell apoptosis as demonstrated by annexin V/PI staining in correspondence with an increase in the BAX/Bcl-2 ratio. A metabonomic study indicated that CA significantly decreased the intracellular concentrations of glutathione and succinate in KKU-213 cells and increased dihydrogen acetone phosphate levels in KKU-100 cells treated with 200 µg/mL of CA compared to the control group. In conclusion, CA induced cellular metabolic changes which are involved in the antioxidant defense mechanism, glycerophospholipid metabolism and the tricarboxylic acid cycle. CA may serve as a potent anticancer agent for CCA treatment by inducing CCA cellular apoptosis.

## 1. Introduction

Cholangiocarcinoma (CCA), also known as bile duct cancer, caused by *Opisthorchis viverrini* (Ov) infection through the consumption of the infected cyprinoid fish, has been recognized as a major public health problem in Northeast Thailand [1,2]. Surgery in combination with adjuvant chemotherapy and radiation therapy is required for increasing the survival rate of CCA patients. Many chemotherapeutic agents such as 5-fluorouracil, cisplatin and gemcitabine have been used to treat CCA patients [3,4]. However, due to the late stage detection of the disease, the treatment is still largely unsuccessful with a low rate of five-year survival [5]. Apoptosis resistance is an important hallmark of CCA, which leads to cell death and cell cycle checkpoints [6]. Recently, there is some evidence that CCA cells can escape apoptosis by increasing or decreasing the expression of anti- or pro-apoptotic proteins [7]. High expression of B-cell lymphoma/leukemia 2 (Bcl-2) in various human cancers mediates the resistance of cancers to a wide range of 5-fluorouracil and cisplatin treatments [8]. An alternative treatment, which is increasingly important in cancer treatment, involves the exploration of new, natural chemotherapy products with apoptosis induction being one of the strategies to inhibit cancer cells.

In general, lignin is an organic cross-linked phenolic polymer found in the cell walls of wood and bark. The biosynthesis of the aromatic monomer of lignins is catalyzed from phenylalanine through the phenylpropanoid biochemical pathway [9]. The structure of lignins is very complex, being polymers of plant cell walls. However, they are formed of only three monolignols: coniferyl (CA), paracoumaryl (PA) and sinapyl (SA) alcohols, of which CA is the predominant monolignol in grasses and softwood. More than two-thirds of the linkages in lignin are β-arylether (β-O-4) linkages. Lignins from different biological sources vary in composition, depending on their particular monomeric units. CA is a colorless, crystalline, organic compound. It is also an intermediate in the biosynthesis of eugenol and coumarin, which show potent anticancer activities [10,11]. Our recent study on metabolic profiling reported a compound from ethanolic *Scoparia dulcis* L extract with two coniferyl alcohols with β-O-4 linkages, which could potentially induce apoptosis of CCA cells [12]. However, few studies have reported the biological activity of coniferyl alcohol on cancer inhibition.

Metabonomics is to characterize many metabolites in cells, tissue extracts and biological fluids (such as urine, plasma, and serum) [13,14]. Moreover, the application of metabolic approaches to the study of precision medicine has become very popular in order to achieve effective cancer treatment outcome [15]. Therefore, metabonomics is of one of the major tools for exploring the metabolic changes induced by disease or treatment, thus enhancing our understanding of disease mechanisms and the mechanism of action for any drug. Moreover, metabonomics assesses the final downstream biological processes of gene transcription and reflects a systemic operation called metabolic phenotypes (also called metabotypes) [16]. There are two main analytical platforms used in metabonomics: mass spectrometry (MS) and nuclear magnetic resonance (NMR) spectroscopy. The advantages of NMR technology include that it is robust and requires minimal sample preparation, as well as providing structural information for metabolite identification. ^1^H NMR-based metabonomics can be used to monitor the metabolic responses of patients to drug treatment to evaluate the efficacy and toxicity of lead compounds [17]. In this study, we investigated the metabolic responses of cholangiocarcinoma cell lines to CA and further studied the potential anticancer mechanisms of CA treatment.

## 2. Materials and Methods

### 2.1. Chemicals and Reagents

Briefly, Trimethylsilylpropionic acid-d4 sodium salt (TSP) was purchased from Cambridge Isotope Laboratory (Tewksbury, Massachusetts, USA). Deuterium oxide (D_2_O) and tetrahydrofuran (THF) were purchased from Merck (Darmstadt, Germany). Dimethyl sulfoxide (DMSO), ferulic acid, benzyl chloride (BnCl), sodium sulfate (Na_2_SO_4_), sodium bicarbonate (NaHCO_3_), lithium aluminium hydride (LiAlH_4_) and sulforhodamine B (SRB) were purchased from Sigma (St Louis, MO, USA). Ham’s F12, penicillin-streptomycin, fetal bovine serum and trypsin were obtained from Thermo Scientific (Grand Island, NY, USA). Enhanced chemiluminescence plus solution (ECL) was purchased by GE healthcare (Buckinghamshire, UK). A General Oxidative Stress Indicator (CM-H_2_DCFDA) and Pierce bicinchoninic acid (BCA) protein assay kit were purchased from Thermo Scientific (Rockford, IL, USA).

The primary antibodies (Ab), including mouse anti-actin Ab, mouse-anti BAX Ab and rabbit-anti Bcl-2 Ab, were purchased from Abcam (Cambridge, UK). The secondary Ab including anti-mouse Ab and anti-rabbit Ab were purchased from GE healthcare (Buckinghamshire, UK) and Sigma (St Louis, MO, USA), respectively.

### 2.2. Cell Lines

The CCA cell lines KKU-100 (JCRB 1568) and KKU-213 (JCRB 1557) were developed by Prof. Banchob Sripa at the Cholangiocarcinoma Research Institute, Khon Kaen University, Thailand under the approval of the Ethics Committee for Human Research, Khon Kaen University (HE571283). Based on the histological classification, KKU-100 was derived from poorly differentiated tubular adenocarcinoma of 65-year-old female patient while KKU-213 was isolated from 58-year-old male patient which characterized with mixed types of papillary and non-papillary adenocarcinoma. Both cell lines were deposited to Japanese Collection of Research Bioresources Cell Bank (JCRB), Osaka, Japan for all complete identification of characteristics. In our study, cell lines were purchased from JCRB and cultured in Ham’s F12 nutrient mixture supplemented with 10% fetal bovine serum and 100 IU/mL of penicillin-streptomycin at 37 °C containing 5% CO_2_ in a humidified incubator.

### 2.3. Coniferyl Alcohol Synthesis

In the present work, we used in house synthesized coniferyl alcohol. The procedure was as previously published [18]. Briefly, ferulic acid (2.0 g, 8.92 mmol, 1.0 equiv.) was dissolved in EtOH and stirred with a drop-wise addition of H_2_SO_4_. The reaction was refluxed for 6 h and left to strand overnight. After removal of the solvent, the organic layer was extracted with ethyl acetate and washed with an aqueous NaHCO_3_ solution and water. Then, ethyl ferulate ((E)-ethyl-3-(4-hydroxy-3-methoxyphenyl) acrylate) was obtained. A suspension of LiAlH_4_ (2.56 g, 67.36 mmol) was prepared with freshly dried THF (200 mL) at 0 °C. Next, the suspension was stirred with a drop-wise addition of ethyl ferulate (10 g, 45 mmol) in THF (40 mL) for 2 h. After that, the reaction was quenched with water, washed with brine and filtered through a celite bed. Then, the filtrate was washed with 200 mL of ethyl acetate and brine. Finally, the organic phase was dried over anhydrous sodium sulfate and evaporated under vacuum to obtain a CA (a light yellow solid).

### 2.4. CCA cell Inhibition Test

Two thousand CCA cells were seeded into 96-well culture plates for 18 h. After that, the medium was removed and renewed with CA stock solution (5 mg/mL) diluted with culture medium. The cells were cultured for 48 or 72 h. Then, the cell viability was determined using a SRB assay [19]. The percentage of cell viability was calculated according to the following formula:

% cell viability = absorbance sample/absorbance negative control or untreated × 100

A half maximum concentration (IC50) was measured as particular of CA concentration that inhibit CCA cell viability by 50%.

### 2.5. The Assessment of Apoptosis Using Flow Cytometry

Firstly, CCA cells (4 × 10^5^ cell/mL) were plated in 6 well plates and incubated for 18–20 h. Then, the CA at concentrations of 50, 100 and 200 µg/mL was added into the cell culture and incubated for 72 h. Cells were trypsinized and resuspended with 200 µL of incubation buffer. Then, apoptotic and necrotic cells were detected using an Annexin-V-FLUOS staining kit (Roche, Penzberg, Germany). Finally, stained cells were immediately detected by flow cytometry (Beckton Dickinson FACSCanto II) and then analyzed by BD FACSDiva software (BD Biosciences, San Jose, CA, USA). The experiment was performed in triplicates.

### 2.6. Western Blot Analysis

CA-treated KKU-100 and KKU-213 cells were collected and lysed using RIPA lysis buffer containing a protease inhibitor cocktail (Cell Signaling Technology, Beverly, Massachusetts, USA). Bicinchoninic acid Protein Assay reagent (Thermo Scientific, Rockford, IL, USA) was used to determine the protein concentration of the extracted cells. Then, 40 µg of protein was dissolved in 4× sodium dodecyl sulfate (SDS) buffer and boiled at 95 °C. Proteins was subjected to 10% SDS-polyacrylamide gel electrophoresis and electrotransferred onto a polyvinylidene fluoride membrane (Merck, Darmstadt, Germany). The membrane was blocked for non-specific binding by incubation with 5% (*w*/*v*) skim milk at RT for 1 hr. The membrane was probed with specific primary Ab as follows: mouse anti-actin Ab (1:10,000), mouse-anti-BAX Ab (1:1000) and rabbit-anti Bcl-2 Ab (1:1000) at 4 °C for 12 h, followed by washing with TBS containing 0.1% Tween 20 (TTBS) 3 times and TBS once. The membrane was then incubated with horseradish peroxidase-conjugated secondary antibody at room temperature for 1 hr. Protein expression was detected using an ECL detection kit and quantified using an ImageQuant Imager (GE Healthcare, Chicago, Illinois, USA). The experiment was done in triplicates.

### 2.7. The Measurement of Intracellular Reactive Oxygen Species (ROS)

Firstly, CCA cells (1 × 10^4^ cell/mL) were seeded into black 96-well plate and incubated for 18 h. Then, CA at concentration of 50, 100 and 200 µg/mL and positive control of 100 µM H_2_O_2_ was added into the cell culture and incubated for 6 h. The CCA cells were washed using 100 µL of 1× phosphate buffer saline (PBS), then incubated the CCA cells with 10 µM of H_2_DCFDA for staining. After incubation in the dark for 45 min, the H_2_DCFDA solution was removed and 1x PBS was added before the immediate detection using microplate reader (Varioskan, Thermo Scientific, Waltham, MA, USA). The experiment was conducted with three replicates.

### 2.8. The Collection of Cells for Metabonomics Analysis

For the study of intracellular metabonomics, CCA cells were seeded in 100 mm cell culture dish for 24 h. After that, the culture medium was removed, then the designed concentrations of CA were added. Ten million CCA cells were collected from three independent experiments. The harvested cells were washed three times with phosphate buffered saline (PBS). They were then resuspended in distilled water before lysis and homogenization by 3 cycles of freezing and thawing in liquid nitrogen. The samples were sonicated and homogenized in 500 µL of water and mixed with 2 mL of a mixture of chloroform/methanol (1:3 *v/v* ratio). Then, the mixture was incubated for 20 min. on ice with a frequency vortex. The samples were centrifuged at 4000× *g* at 4 °C for 20 min. The upper aqueous phase containing water soluble metabolites was collected into a new 1.5 mL tube. The aqueous phase was evaporated to a dry state in a SpeedVac at 4 °C, after which the tube was kept at −80 °C for NMR metabonomics analysis.

### 2.9. ^1^H-NMR Based Metabonomics Analysis

Aqueous cell extracts and cell culture media were diluted with D_2_O buffer (1.5 M KH_2_PO_4_, 2 mM NaN_3_ and 1% TSP), then vortexed for 1 min. at room temperature. After 20 min. ultrasonication on ice, the mixture was centrifuged at 10,000× *g* at 4 °C for 10 min. The supernatant was transferred into a 5 mm NMR tube. Proton NMR spectra were acquired using a 400 MHz NMR spectrometer (Avance, Bruker BioSpin GmbH, Rheinstetten, Germany) at a temperature of 26.8 °C. The Carr-Purcell-Meiboom-Gill sequence (CPMG) pulse program was applied which was a presaturation pulse sequence for water suppression from the standard pulse sequence in the spectrometer library. The total of 64 scans were collected into 32  K data points with a relaxation delay of 4 sec. After NMR data acquisition, chemical shift referencing, baseline correction, and phasing were processed using MestReNova (Mestrelab Research, Escondido, CA, USA) software. Then, all spectra were aligned, normalized and the scaling aligned in MATLAB software (Mathworks Inc., Natick, MA, USA). To confirm the metabolite assignment of correlated resonances, statistical total correlation spectroscopy (STOCSY) was employed and searched against online metabolite databases such as human metabolome database (HMDB), biological magnetic resonance data bank (BMRD) and Chenomx software (Chenomx Inc., Edmonton, Canada).

### 2.10. Statistical Analysis

The half inhibitory effects (IC50) were expressed as a mean ± SD. In order to compare the percentage of apoptotic cells, significant differences were evaluated using one-way analysis of variance (ANOVA) followed by Duncan’s multiple-range test (*p*-value < 0.05) with GraphPad software (GraphPad Software, San Diego, CA, USA). To extract maximum information on discriminant compounds from the spectra, multivariate analysis was performed using MATLAB (Mathworks, Natick, MA, USA) and SIMCA-P+ (Umetrics, Umea, Sweden) software. A color code visualized the concentration differences between each group in the OPLS-DA model which corresponds to the squared correlation coefficient in the OPLS-DA loadings as described by Cloarec et al. (2005) [20]. Moreover, the integral area of the peaks identified in ^1^H NMR, which corresponded to individual metabolites, was performed to quantify the absolute concentrations of these metabolites.

## 3. Results

### 3.1. CA Inhibits CCA Cell Growth in a Dose Dependent Manner

**Figure 1:**
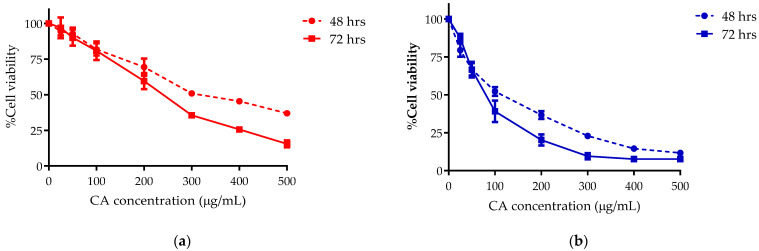
The cell viability of (**a**) KKU-100 and (**b**) KKU-213 after CA treatment for 48 and 72 h; data represents the mean  ±  standard deviation of three independent experiments.

KKU-100 and KKU-213 cells responded to the inhibitory effect of CA in a dose dependent manner. The IC50s of CA on KKU-100 were 361.87 ± 30.58 and 268.27 ± 18.61 μg/mL after 48 and 72 h exposure, respectively, and on KKU-213 184.37 ± 11.15 and 151.03 ± 24.99 μg/mL after 48 and 72 h exposure, respectively. Therefore, CA was shown to have more inhibitory effect on KKU-213 cell growth compared with KKU-100 (Figure 1). The IC50 of CA was slightly higher than that of the ethanolic extract of Scoparia dulcis (ESD) on KKU-213 of both treatments for 48 and 72 h compared to our previous study [12].

### 3.2. Assessment of CA-Induced Apoptosis Effects

**Figure 2:**
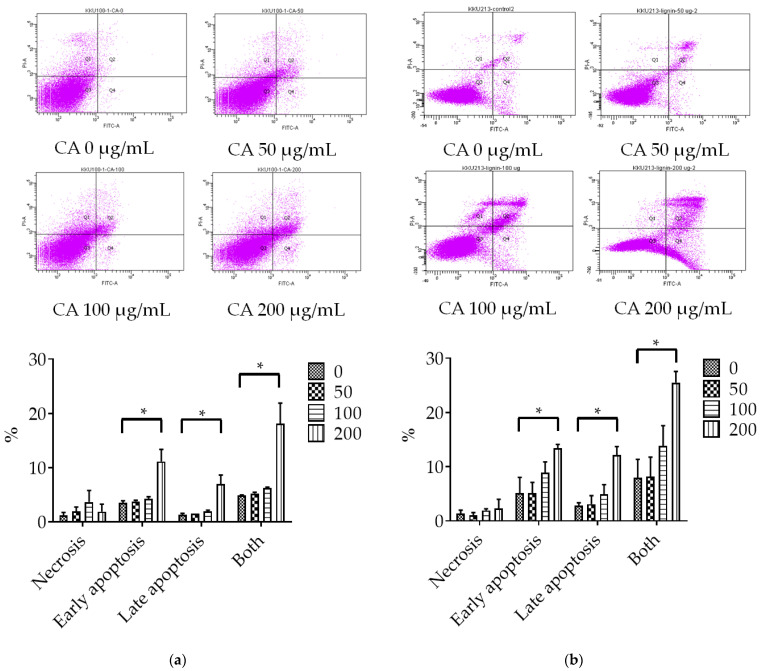
Flow cytometric analysis of apoptotic cells of (**a**) KKU-100 and (**b**) KKU-213 cell lines stained with propidium iodide and FITC-Annexin V after treatment with CA for 48 h. (Data was presented in triplicate.* = statistically significant; *p*-value < 0.05).

**Figure 3:**
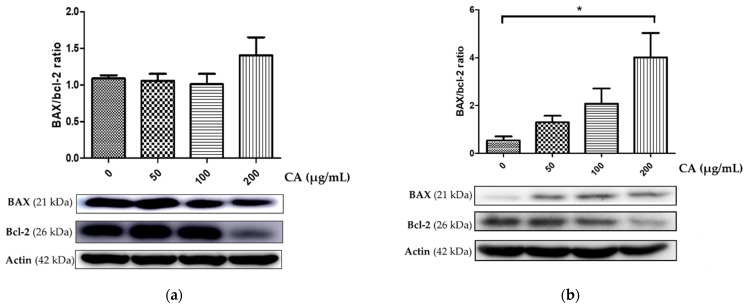
BAX and Bcl-2 protein expression of (**a**) KKU-100 and (**b**) KKU-213 after treatment with CA for 48 h. (* = statistically significant; *p*-value < 0.05).

To determine apoptotic cell death, different concentrations of CA (0, 50, 100 and 200 μg/mL) were added to KKU-100 and KKU-213 cells. The populations of early- and late-stage apoptotic cells were significantly increased in both KKU-100 and KKU-213 cell lines after treatment with 200 µg/mL of CA for 48 h (Figure 2). CA-treated KKU-213 cells showed a significant increase in BAX protein expression and a decrease in Bcl-2 expression in a dose dependent manner (Figure 3). However, the ratio of BAX/Bcl-2 protein expression compared to the control group for KKU-100 cells was not statistically significant.

### 3.3. Metabonomics of CCA Cells Treated with CA

**Figure 4:**
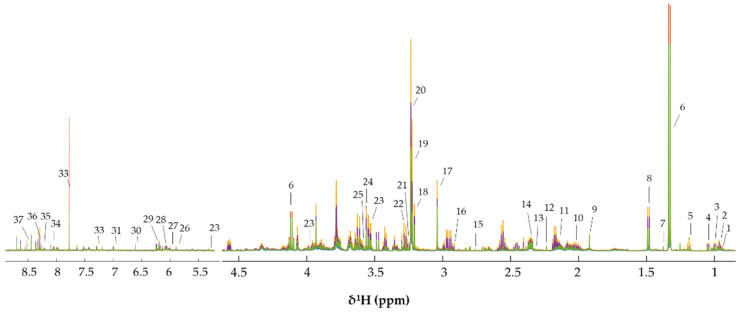
^1^H-NMR spectra of the intracellular metabolites of KKU-100 and KKU-213 cells. Key metabolites: (1) isovalerate (2) leucine (3) isoleucine (4) valine (5) ethanol (6) lactate (7) dimethylmalonic acid (8) alanine (9) acetate (10) homocysteine (11) proline (12) oxaloacetate (13) succinate (14) glutamate (15) dimethylamine (16) glutathione (17) creatine (18) choline (19) phosphocholine (20) carnitine (21) betaine (22) taurine (23) glucose (24) glycine (25) dihydroxyacetone phosphate (26) uracil (27) uridine (28) NAD^+^ (29) ATP (30) fumarate (31) tyrosine (32) phenylalanine (33) guanine (34) guanosine (35) adenine (36) inosine (37) formate.

**Figure 5:**
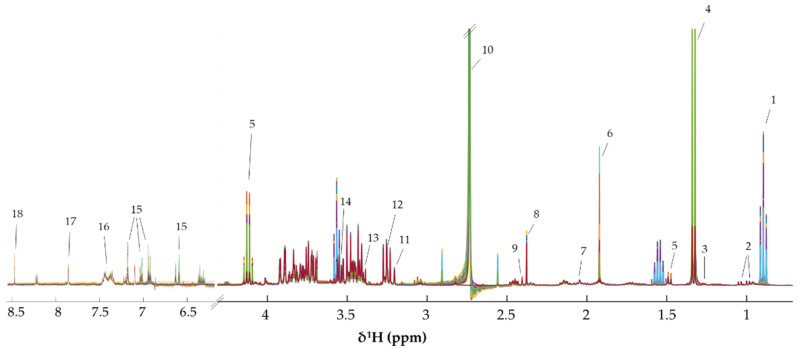
^1^H-NMR spectra of extracellular metabolites of KKU-100 and KKU-213 cells. Key metabolites: (1) L-alpha-aminobutyric acid (2) valine (3) methylmalonate (4) lactate (5) alanine (6) acetate (7) glutamine (8) pyruvate (9) succinate (10) dimethylamine (11) choline (12) arginine (13) glycine (14) glucose (15) coniferyl alcohol (16) phenylalanine (17) hypoxanthine (18) formate.

To explore the metabolic changes in KKU-100 and KKU-213 cells induced by CA treatment, intracellular (cell aqueous extract) and extracellular (cell cultured media) metabolites were identified as shown in Figure 4 and Figure 5, respectively. Amino acid, amines, sugars, short chain fatty acids (SCFAs) and choline were present in the spectra of intracellular KKU-100 and KKU-213, whereas, amino acids, glucose, SCFAs and CA were presented in the spectra of the extracellular metabolites of KKU-100 and KKU-213 in the culture media. The corresponding peaks of intracellular and extracellular metabolites are shown in Supplementary Tables S1 and S2, respectively.

**Figure 6:**
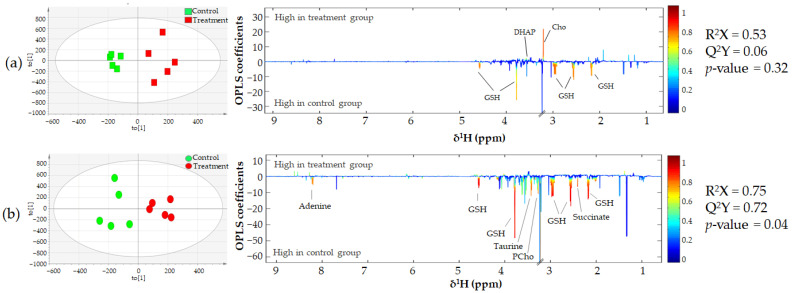
OPLS-DA scores (left panel) and loadings (right panel) plots displaying significantly changed intracellular metabolite levels after treatment with or without 200 µg/mL CA on (**a**) KKU-100 and (**b**) KKU-213 cell lines. The positive peaks of the OPLS-DA loading plot indicate metabolites higher in the treatment group, whereas the negative peaks represent metabolites higher in the control group. The color of the peaks corresponds to the correlation coefficients in the discrimination model.

Multivariate statistical analysis was performed based on Pareto-scaled data to explore the metabolic changes induced by CA. In Figure 6, a goodness of fit (R^2^X) and a goodness of prediction (Q^2^Y) for the KKU-213 model was used to evaluate the quality of the statistical models for which a value above 0.5 was considered acceptable for a classification model for biological samples [21]. In addition, the permutation *p*-value < 0.05 was used to cross validate of the model. The OPLS-DA model identified intracellular metabolites in KKU-213 cells, such as glutathione (GSH), succinate, taurine, adenine and phosphocholine (PCho), which were decreased in the 200 µg/mL CA treatment compared to the control group. Moreover, relative levels of metabolite, represented by the area under the peak of the identified metabolites, was analyzed using one-way analysis of variance (ANOVA) followed by Duncan’s multiple-range test as summarized in Figure S3. In KKU-213 cells, the univariate results were similar in OPLS-DA model. The level of dihydroxyacetone phosphate (DHAP) and choline (Cho) was increased in KKU-100 cell treatment with CA in a dose dependent manner. ATP levels likely increased in KKU-213 in a dose dependent manner, however, these were not statistically significant. In KKU-100 cells, the levels of glutathione were significantly decreased in the 200 µg/mL of CA treatment group compared to the control group. Moreover, the intracellular ROS level of CCA treated cells was significantly increased according to the decrease of GSH level in CA dose dependent manner (Supplementary Figure S6).

**Figure 7:**
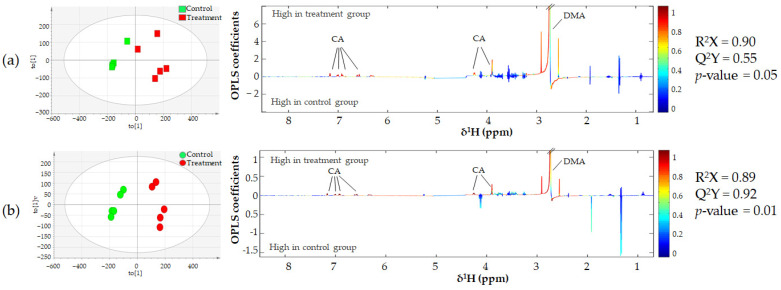
OPLS-DA scores and loading plots displaying significant extracellular metabolites after treatment with or without 200 µg/mL CA on (**a**) KKU-100 and (**b**) KKU-213 cell lines. The positive peaks of the OPLS-DA loading plot indicate metabolites higher in the treatment group, whereas the negative peaks represent metabolites higher in the control group. The color of the peaks corresponds to the correlation coefficients in the discrimination model.

The cultivation of KKU-100 and KKU-213 cells utilized essential (arginine and valine) and non-essential (glycine and glutamine) amino acids, hypoxanthine, glucose, and choline after incubation for 48 h. Moreover, it released end products from central carbon metabolism, such as lactate and acetate, as shown in Supplementary Figure S4. The CA treatment groups showed the excretion of dimethylamine (DMA) into the cell culture media compared to the control group as shown in Figure 7. Likewise, univariate analysis revealed that the level of hypoxanthine increased in CA in a dose dependent manner in KKU-213 after treatment with CA, whereas the glutamine level decreased in KKU-100 and KKU-213 cell culture media (Supplementary Figure S5).

## 4. Discussion

**Figure 8:**
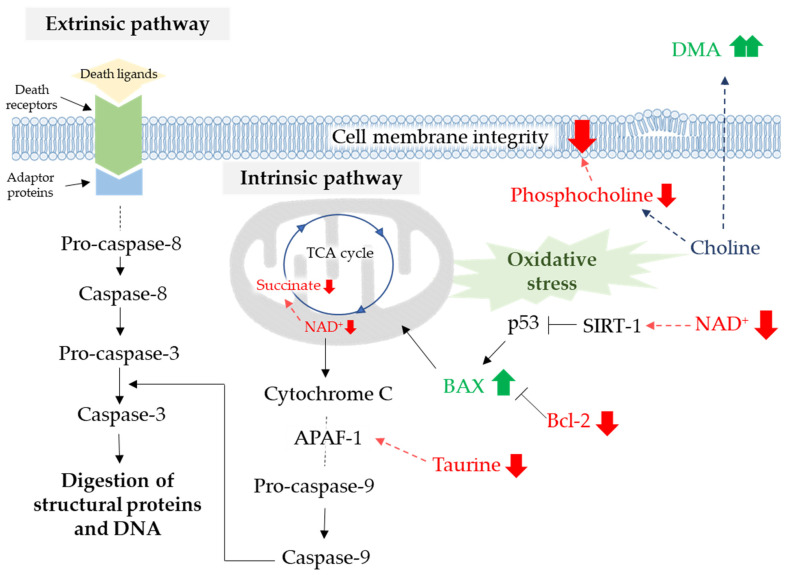
The apoptotic pathways and their relevant metabolites. The red arrow represents a decreased level and the green arrow an increased level compared to the control.

Herbal plants provide a wide range of different bioactive compounds, including phenolics, vitamins, minerals, and fibers. These compounds have been reported to possess important biological properties, such as anticancer, antiviral, antioxidant and anti-inflammatory activities [22]. Phenolics are a variety chemical classes with one or more hydroxy group in their structure [23]. Lignins are present in most phenolics in the structure of cell walls. A recent study found that low-molecular-weight monomers derived from hydroxycinnamic acids and guaiacyl units are responsible for the antioxidant properties of lignins [24]. The precise chemical structure of lignin is not known, because of its complex polymeric nature and due to the degree of random coupling of the monolignols involved in the arrangement of the macromolecule. There are no reports of the effect of monolignols on human health; therefore, this study investigated the effect CA on cholangiocarcinoma. CA is the most common monolignol found in nature and induces CCA cell inhibition through the induction of apoptosis as summarized in Figure 8.

Apoptosis, programmed cell death, can be induced by many promising anticancer agents [25]. High expression of Bcl-2 mediates the resistance to cisplatin in various human cancers [8]. In addition, the upregulated expression of glutathione contributes to CCA cell resistance to anticancer agents [26]. Western blot analysis revealed that 200 µg/mL of CA triggered apoptotic cell death in KKU-213 by significantly elevating the ratio of BAX/Bcl-2, but this did not occur in KKU-100 cells. Moreover, CA induced apoptosis in KKU-100 and KKU-213 cells in a dose dependent manner as observed by annexin V and PI staining. These findings suggest that CA may reduce the integrity of the CCA cell membranes. Annexin V-FITC was stained by binding with phosphatidylserine in the outer cell membrane due to its flip from the inner side of the plasma membrane. Therefore, at sufficient doses of CA, KKU-213 exhibits a molecular switch after exposure to CA that can lead to the induction of the intrinsic apoptosis pathway [27].

Metabonomics was used to access the molecular mechanisms of this potential anticancer agent to induce cancer cell death. We performed cell extraction following the protocol of Bligh and Dyer (1959) [28]. The PCA of the ^1^H NMR spectral data revealed an unclear separation in both cell lines treated with CA, as well as the controls, therefore, supervised OPLS-DA was applied to augment the classification performance between each paired sample group. In our study, the parameters of the OPLS model classification of the KKU-213 cell extract samples were R^2^X > 0.5 and Q^2^Y > 0.5 in the 200 µg/mL of the CA treatment group compared to the control group, which demonstrated an acceptable goodness of fit and predictability [29]. We found a decrease in adenine in KKU-213 cells treated with CA, which might effect a lack of essential biomolecules such as nicotinamide adenine dinucleotide (NAD^+^) and flavin adenine dinucleotide (FAD). However, Han et al. demonstrated that adenine inhibited cancer proliferation by cell cycle arrest and apoptosis induction [30]. NAD^+^ is a co-enzyme that mediates various cellular metabolic processes including glycolysis, the tricarboxylic acid (TCA) cycle and oxidative phosphorylation [31]. Therefore, a lack of NAD^+^ is a cause of the decreasing succinate levels found in our study. In addition, CA significantly impacted on TCA cycle metabolism which was significantly affected by the decrease of succinate. CA induced metabolic changes in a dose dependent manner as indicated in the OPLS-DA model. Moreover, DNA damage was not repaired, and the decrease of cellular NAD^+^ levels impairs sirtuin (SIRT) activities leading to tumor protein 53 (p53) activation in apoptotic cells under stress conditions [32]. However, CA has been shown to have a lower inhibition sensitivity in KKU-100 cells due to their TP53 mutations [33]. This observation is similar to our finding that NAD^+^ and succinate levels decline in the CA treatment group. ATP plays a critical role in early apoptosis as it interacts with apoptotic protease activating factor 1 (Apaf-1) before activation of the caspase cascade pathway [34]. Previous reports suggested that taurine induced apoptosis in many types of cancers [35,36]. However, the exposure of taurine can suppress the apoptosis in cardiomyocyte by inhibition interaction between apaf-1 and caspase-9 to form apoptosomes [37]. The decreased taurine levels were observed as KKU-213 underwent apoptosis. Glutathione depletion is important in cellular defense against ROS, especially in apoptosis, indicated by the fact that KKU-100 and KKU-213 underwent activation of the apoptotic signaling cascade [38]. Thus, CA can disturb the antioxidant defense mechanism in KKU-213 cells. Choline is an essential nutrient that is important to cellular function and acts as the precursor of structural components of cell membranes. Therefore, the dose-dependent decrease in the levels of choline and phosphocholine with CA in KKU-100 and KKU-213 cells, respectively, could be associated with the lack of membrane phosphatidylcholine and sphingomyelin and the accumulation of ceramide and diacylglycerol, promoting apoptosis [39].

For extracellular metabolites, we observed an increase in the levels of lactate, acetate and formic acid in cell culture media as metabolic end products. However, these metabolites were not significantly different between the control and CA treatment groups. DMA is formed from trimethylamine (TMA), which, in turn, is a breakdown product of choline [40]. Our study showed elevated levels of DMA in KKU-213 and KKU-100 cells treated with CA in a dose dependent manner. As DMA is toxic to the cells, it might be excreted into the cell culture media. KKU-100 cells showed a decrease in the level of glutamine as in a similar report on emodin treatment for HepG2 cells [41]. The phosphorylation of ribose from inosine generates hypoxanthine by purine nucleoside phosphorylase (PNP) in a salvage pathway. These metabolites were also found in the apoptotic cell supernatant caused by irradiation and heat in melanoma cells [42], which agrees with the increase in their levels after treatment with CA in KKU-213 cells. Therefore, hypoxanthine might be a marker for the occurrence of apoptotic cells.

Nevertheless, the inhibitory mechanisms of CA on the healthy cells possibly affected by this compound could be similar to CCA cells. Several evidence suggested that the specific targeting cancer cell redox homeostasis provides a therapeutic promising to enhance cytotoxicity on cancer cells. Due to the high intracellular ROS level of cancer cells, CA was preferentially sensitizing the CCA cell apoptosis indicated by the decreased GSH level observed in CCA cells. Particularly, the use of the CA monomer, which was developed for lignin nanoparticles (LNPs), may offer great benefits to provide non-toxicity, flexibility, and stability properties. A previous study demonstrated that LNPs were able to control the release in gastric pH-dependent and also exhibited controlled release properties in animal models with less toxicity on normal cells [43,44]. Moreover, the breakdown of lignin to monomer might be synergizing the efficacy of anticancer drugs.

In this study we observed the effect of the CA on the metabolic changes in CCA cells. Further study should increase, using other high throughput techniques, our understanding of the molecular mechanisms related to apoptosis induction. Moreover, nanoparticle technology with a standard cancer regimen should be used to develop a suitable release mechanism for enhancing drug sensitivity against CCA cells.

## 5. Conclusions

We showed that the inhibitory effect of CA on KKU-213 cell growth was greater than for KKU-100. In addition, the BAX/Bcl-2 ratio was increased in the CA treated groups in a dose dependent manner. However, there was no change in apoptotic protein expression in KKU-100. Intracellular metabolic changes were observed by the disturbed antioxidant defense, TCA cycle and choline metabolism. Moreover, the excretion of a toxic compound in choline metabolism, e.g., DMA, was observed in the cell culture media Therefore, CA could be developed into a new chemotherapeutic agent for the prevention or treatment of CCA.

## Data Availability

Not applicable.

## Supplementary Materials

The following are available online at https://www.mdpi.com/2218-273X/11/3/476/s1, Table S1: The identification of intracellular metabolites detected in the metabonomic study of cell extract, Table S2: The identification of extracellular metabolites detected in the metabonomic studied of cell cultured media, Figure S1: Principal component analysis of intracellular metabolites of CCA cells after treatment with or without CA, Figure S2: Principal component analysis of extracellular metabolites of CCA cells after treatment with or without CA, Figure S3: Comparison of significantly changed intracellular metabolite concentrations, Figure S4: OPLS-DA comparison of metabolites in the cell free media and cell cultured media. Figure S5: Comparison of significantly changed extracellular metabolites concentrations. Figure S6: The measurement of intracellular ROS level.
