# Supplementary material for: Metabolic Changes of Cholangiocarcinoma Cells in Response to Coniferyl Alcohol Treatment
Source: Biomolecules. 2021 Mar 22;11(3):476. doi: 10.3390/biom11030476 (PMC8004792; doi:10.3390/biom11030476)
Supplement: Supplementary file 1 [file biomolecules-11-00476-s001.pdf]

Supplementary Files

Table S1. The identification of intracellular metabolites detected in the metabonomic study of cell extracts. The metabolites were identified by observing the corresponding peak using the STOCSY method and then searching public databases such as HMDB, BMRB and FooDB. ^{1}H-NMR data are measured in ppm.

Potential metabolites	Chemical shift values
Isovalerate	δ 0.93 (d)
Leucine	δ 0.95 (t), δ 0.99 (d), δ 1.04 (d), δ 1.53 (s), δ 1.65 (m), δ 1.89 (d)
Isoleucine	δ 1.01(d)
Valine	δ 0.98 (d), δ 1.03 (d), δ 2.12 (m)
Ethanol	δ 1.17 (t), δ 3.64 (q)
Lactate	δ 1.32 (d), δ 4.09 (q)
Dimethylmalonic acid	δ 1.43 (s)
Alanine	δ 1.47 (d)
Acetate	δ 1.92 (s)
Homocysteine	δ 2.142(m), δ 2.517(m), δ 3.874 (dd)
Proline	δ 2.040(m), δ 2.339(m), δ 3.752(m), δ 4.125(q)
Oxaloacetate	δ 2.386(s)
Succinate	δ 2.406(s)
Glutamate	δ 2.11(m), δ 2.44 (m)
Dimethylamine	δ 2.73 (s)
Glutathione	δ 2.15 (m), δ 2.52 (m), δ 2.92 (m), δ 3.75 (m), δ 4.20
Creatine	δ 3.04 (s), δ 3.93 (s)
Choline	δ 3.20 (s)
Phosphocholine	δ 3.22 (s), δ 3.56 (m)
Carnitine	δ 2.34 (m), δ 3.23 (s)
Betaine	δ 3.25 (s)
Taurine	δ 3.26 (t), δ 3.41 (t)
Glucose	δ 3.23 (m), δ 3.39 (m), δ 3.89 (dd), δ 5.24 (d)
Glycine	δ 3.56 (s)
Dihydroxyacetone	δ 3.58 (s)
Uracil	δ 5.80 (d), δ 7.54 (d)
Uridine	δ 5.90 (s), δ 7.87 (d)
NAD^{+}	δ 6.04 (d), δ 6.09 (d), δ 8.18, δ 8.43 (s), δ 8.83, δ 9.35 (s)
ATP	δ 6.14 (d), δ 8.27 (s), δ 8.54 (s)
Fumarate	δ 6.52 (s)
Tyrosine	δ 6.90 (d), δ 7.19 (d)
Phenylalanine	δ 7.33 (d), δ 7.38 (t), δ 7.42 (m)
Guanine	δ 7.68 (s)
Guanosine	δ 5.90 (d), δ 8.00 (s)
Adenine	δ 8.20 (s), δ 8.22 (s)
Inosine	δ 6.10 (d), δ 8.24 (s), δ 8.35 (s)
Formic acid	δ 8.46 (s)

Table S2. The identification of extracellular metabolites detected in the metabonomic study of cell culture media. The metabolites were identified by observing the corresponding peak using the STOCSY method and then searching public databases such as HMDB, BMRB and FooDB. ^{1}H-NMR data are measured in ppm.

**Metabolites**	**Chemical shift values**
L-alpha-aminobutyric acid	δ 0.88 (t), δ 1.51 (m), δ 3.549 (t)
Valine	δ 0.98 (d), δ 1.03 (d)
Methylmalonate	δ 1.26 (d)
Lactate	δ 1.32 (d), δ 4.09 (q)
Alanine	δ 1.49 (d)
Acetate	δ 1.92 (s)
Glutamine	δ 2.12 (m), δ 2.43 (m)
Pyruvate	δ 2.38 (s)
Succinate	δ 2.41 (s)
Dimethylamine	δ 2.73 (s)
Choline	δ 3.21 (s)
Arginine	δ 1.63 (m), δ 1.91 (m), δ 3.25 (t), δ 3.77 (m)
Glucose	δ 3.23 (m), δ 3.39 (m), δ 3.89 (dd), δ 5.24 (d)
Glycine	δ 3.56 (s)
Coniferyl alcohol	δ 3.77 (t), δ 4.07 (d), δ 6.57 (d), δ 6.87 (d), δ 6.91 (d), δ 6.99 (d)
Phenylalanine	δ 7.32 (m)
Hypoxanthine	δ 8.20 (d)
Formic acid	δ 8.46 (s)

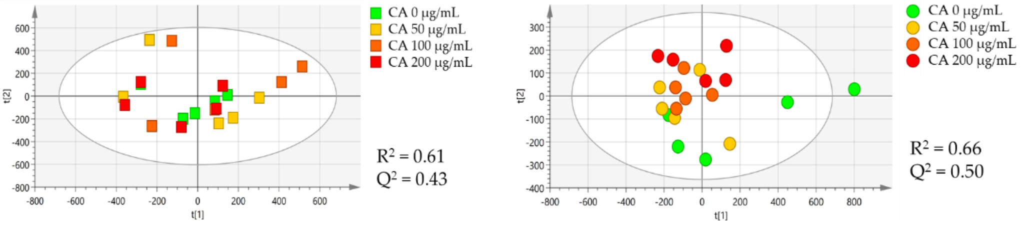

(a) (b)

Figure S1. Principal component analysis of intracellular metabolites of (a) KKU-100 and (b) KKU-213 cells after treatment with or without CA

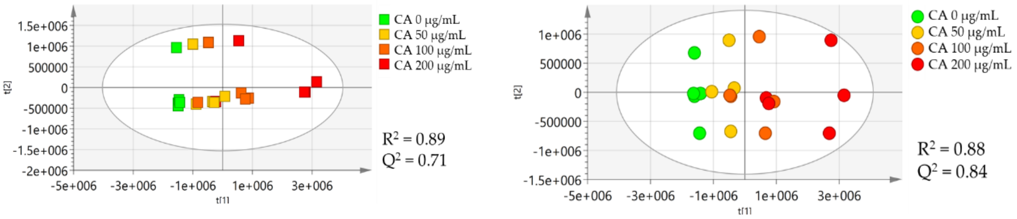

(a) (b)

Figure S2. Principal component analysis of extracellular metabolites of (a) KKU-100 and (b) KKU-213 cells after treatment with or without CA

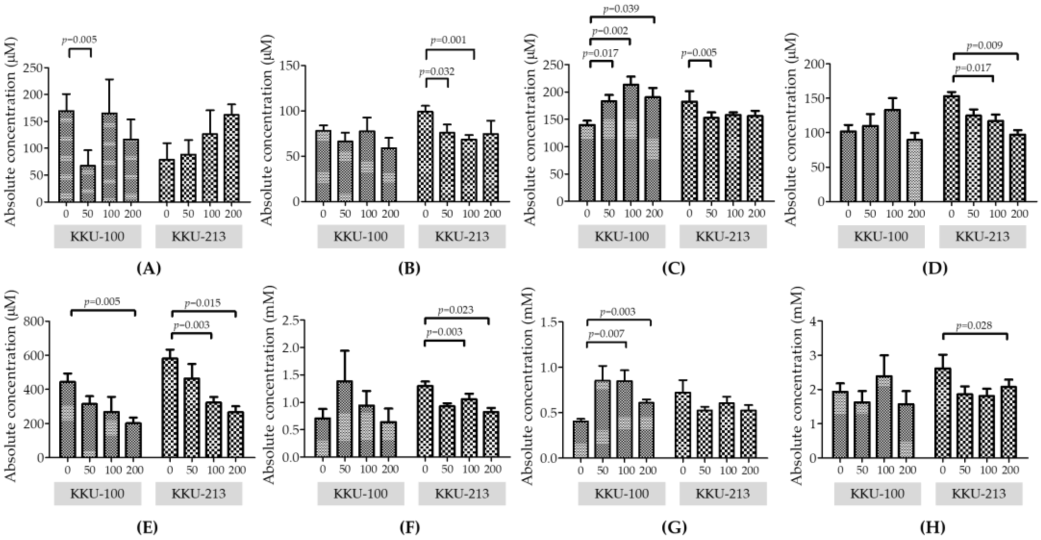

Figure 3. Comparison of significantly changed intracellular metabolite concentrations. (A) ATP (B) NAD+ (C) Dihydroxyacetone phosphate (D) Succinate (E) Glutathione (F) Phosphocholine (G) Choline (H) Carnitine

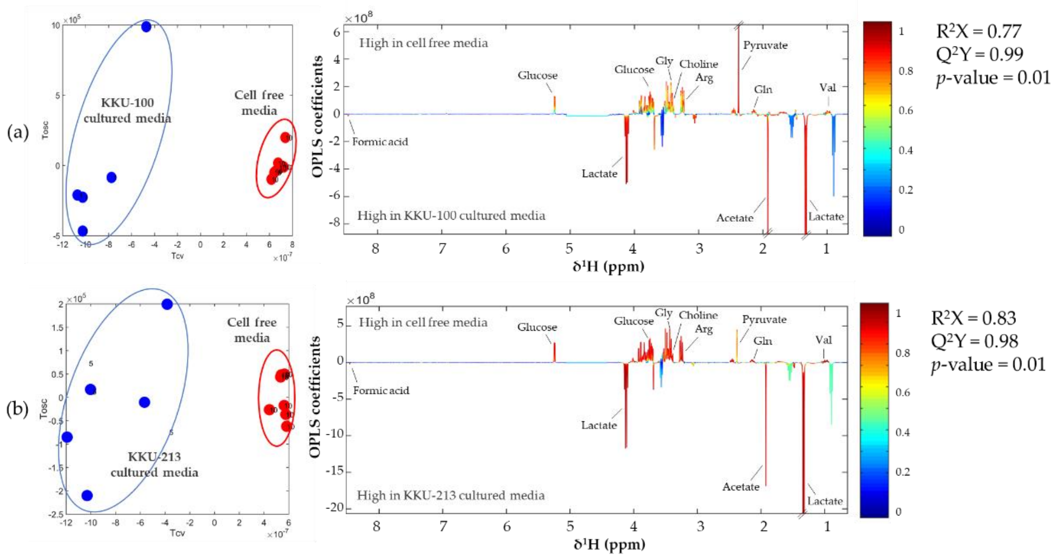

Figure S4. OPLS-DA comparison of metabolite in the cell free media and cell cultured media.

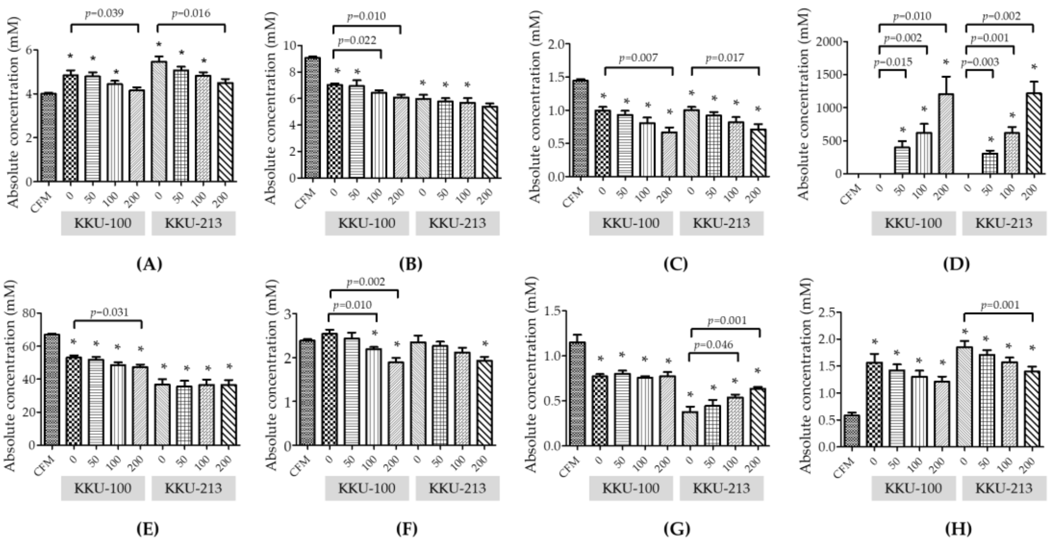

Figure S5. Comparison of significant changes in extracellular metabolite concentrations (* statistical significantly compared to cell free media (CFM); *p*-value < 0.05) (A) Alanine (B) Glutamine (C) Arginine (D) DMA (E) Glucose (F) Succinate (G) Hypoxanthine (H) Formic acid

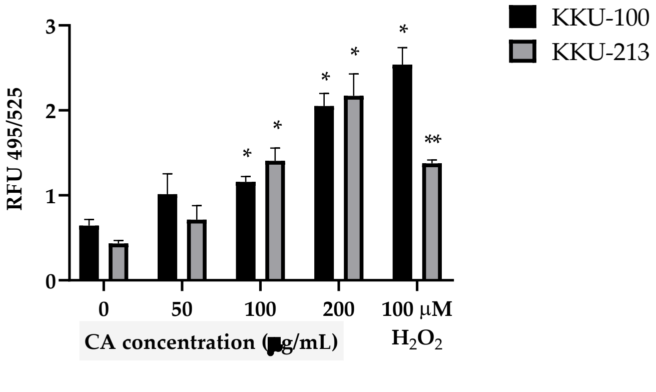

Figure S6. The measurement of intracellular ROS level. Data are plotted for three independent replicates. 100 μM is positive control (* p-value < 0.05, ** p-value < 0.001).
